# A complete mitochondrial genome for fragrant Chinese rosewood (*Dalbergia odorifera*, Fabaceae) with comparative analyses of genome structure and intergenomic sequence transfers

**DOI:** 10.1186/s12864-021-07967-7

**Published:** 2021-09-18

**Authors:** Zhou Hong, Xuezhu Liao, Yuanjun Ye, Ningnan Zhang, Zengjiang Yang, Weidong Zhu, Wei Gao, Joel Sharbrough, Luke R. Tembrock, Daping Xu, Zhiqiang Wu

**Affiliations:** 1 State Key Laboratory of Tree Genetics and Breeding, Research Institute of Tropical Forestry, Chinese Academy of Forestry, Guangzhou, 510520 China; 2 Shenzhen Branch, Guangdong Laboratory for Lingnan Modern Agriculture, Genome Analysis Laboratory of the Ministry of Agriculture, Agricultural Genomics Institute at Shenzhen, Chinese Academy of Agricultural Sciences, Shenzhen, 518120 China; 3 Guangdong Provincial Key Lab of Ornamental Plant Germplasm Innovation and Utilization, Environmental Horticulture Research Institute, Guangdong Academy of Agricultural Sciences, Guangzhou, 510640 China; 4 College of Plant Science & Technology, Huazhong Agricultural University, Wuhan, 430070 Hubei China; 5 Biology Department, New Mexico Institute of Mining and Technology, Socorro, NM 87801 USA; 6 Department of Agricultural Biology, Colorado State University, Fort Collins, CO 80523 USA

**Keywords:** Plant mitochondrial genome, Chloroplast genome, Horizontal gene transfer, Repetitive DNA, Phylogeny, Threatened species

## Abstract

**Background:**

*Dalbergia odorifera* is an economically and culturally important species in the Fabaceae because of the high-quality lumber and traditional Chinese medicines made from this plant, however, overexploitation has increased the scarcity of *D. odorifera*. Given the rarity and the multiple uses of this species, it is important to expand the genomic resources for utilizing in applications such as tracking illegal logging, determining effective population size of wild stands, delineating pedigrees in marker assisted breeding programs, and resolving gene networks in functional genomics studies. Even the nuclear and chloroplast genomes have been published for *D. odorifera*, the complete mitochondrial genome has not been assembled or assessed for sequence transfer to other genomic compartments until now. Such work is essential in understanding structural and functional genome evolution in a lineage (Fabaceae) with frequent intergenomic sequence transfers.

**Results:**

We integrated Illumina short-reads and PacBio CLR long-reads to assemble and annotate the complete mitochondrial genome of *D. odorifera*. The mitochondrial genome was organized as a single circular structure of 435 Kb in length containing 33 protein coding genes, 4 rRNA and 17 tRNA genes. Nearly 4.0% (17,386 bp) of the genome was annotated as repetitive DNA. From the sequence transfer analysis, it was found that 114 Kb of DNA originating from the mitochondrial genome has been transferred to the nuclear genome, with most of the transfer events having taken place relatively recently. The high frequency of sequence transfers from the mitochondria to the nuclear genome was similar to that of sequence transfer from the chloroplast to the nuclear genome.

**Conclusion:**

For the first-time, the complete mitochondrial genome of *D. odorifera* was assembled in this study, which will provide a baseline resource in understanding genomic evolution in the highly specious Fabaceae. In particular, the assessment of intergenomic sequence transfer suggests that transfers have been common and recent indicating a possible role in environmental adaptation as has been found in other lineages. The high turnover rate of genomic colinearly and large differences in mitochondrial genome size found in the comparative analyses herein providing evidence for the rapid evolution of mitochondrial genome structure compared to chloroplasts in Faboideae. While phylogenetic analyses using functional genes indicate that mitochondrial genes are very slowly evolving compared to chloroplast genes.

**Supplementary Information:**

The online version contains supplementary material available at 10.1186/s12864-021-07967-7.

## Introduction

The genus *Dalbergia* includes over 250 species (Fabaceae, tribe Dalbergieae), of which most are woody trees, shrubs, and lianas found in the tropical and subtropical areas of the world [1] (Hong Z 2021, submitted). Within this genus, some tree species are used to produce high-value fragrant wood and traditional medicines. The rarity of these trees in the wild and the high quality of the wood has resulted in extremely high prices being paid for such logs, and the development of a lucrative international illegal lumber trade to supply demand [1]. As such, developing genomic resources for the rarest and most highly valued species is essential for tracking illegal logging and developing conservation genomic based strategies for reintroduction and preservation of threatened wild populations.

Among the *Dalbergia* used for timber, *D. odorifera* T. Chen (previously *D. hainanensis* Merr. et Chun) is considered as one of the most valuable trees producing a high-quality fragrant rosewood. In addition to being used for timber, *D. odorifera* is listed in the Chinese Pharmacopoeia as “JiangXiang” and widely used to treat blood stagnation syndrome, ischemia, and other diseases, with similar uses noted in Korea [2]. Given the importance of *D. odorifera,* it was the first *Dalbergia* species for which the entire nuclear genome was sequenced [3] and will be used as a model species to study the genetics of rosewood heartwood formation. Similarly, the chloroplast genome was recently completed and published (Hong Z 2021, submitted). Even the chloroplast and nuclear genomes provide essential data for numerous different applications [4], the entire cellular genomic content remains incomplete until the mitochondrial genome is sequenced and assembled.

As has been thoroughly documented, the mitochondria are essential for several metabolic processes such as cellular respiration and ATP synthesis [5]. The evolution of mitochondrial genomes since the acquisition of alpha-proteobacteria into early eukaryotic cells (endosymbiosis) has involved numerous structural rearrangements and gene transfers to the nuclear genome [6–10]. In plants, and unlike most animals, the mitochondrial genomes can vary by orders of magnitude in size and be partitioned in numerous structural arrangements including multiple circular chromosomes [10, 11]. The variability of plant mitochondrial genomes can even fluctuate greatly within a species [11, 12]. Structural rearrangements in mitochondrial genes can have important outcomes for survival as in the case of gene chimerism resulting in cytoplasmic male sterility [13]. Another important feature of plant mitochondrial genome evolution is gene or sequence transfer to the nuclear and chloroplast genomes [8, 14–16]. During the last one billion years of evolution, plant mitochondrial genomes have been dramatically reshaped from the ancestral alpha-proteobacteria genome. Over time, most of the mitochondrial genes and nonfunctional fragments have been lost or transferred to the nuclear genome [17], and this process is still ongoing today. In addition to intergenomic transfer of genes or gene fragments within a cell, recent studies have shown that entire plastids can be transferred from cell to cell and from individual to individual when tissue grafts are created [18]. This indicates not only the fragments are able to be transferred between cellular compartments but the entire genomes could be transferred across species boundaries. Tracing intergenomic transfer is therefore essential to understand the evolution of plant mitochondrial genomes. In order to better understand the evolution and divergence of plant lineages, all three genomes should be properly characterized to assess how transfers and mutations in one genome have altered the coordination of essential metabolic processes throughout the cell and across speciation events.

Based on the importance of the plant mitochondrial genome in the understanding of evolution and the use of *D. odorifera* as a model species, we conducted the followings: 1) using Illumina short-reads and Pacific Biosciences single-molecule real-time long sequencing reads [3], we assembled and annotated the full mitochondrial genome of *D. odorifera,* 2) compared the mitochondrial genome from *D. odorifera* with other published Fabaceae species to elucidate what changes have occurred across the family, and 3) compared sequence transfer from the chloroplast and mitochondria to the nuclear genome to assess how they differ from each other.

## Results

### The *D. odorifera* mitochondrial genome assembly and annotation

**Fig. 1:**
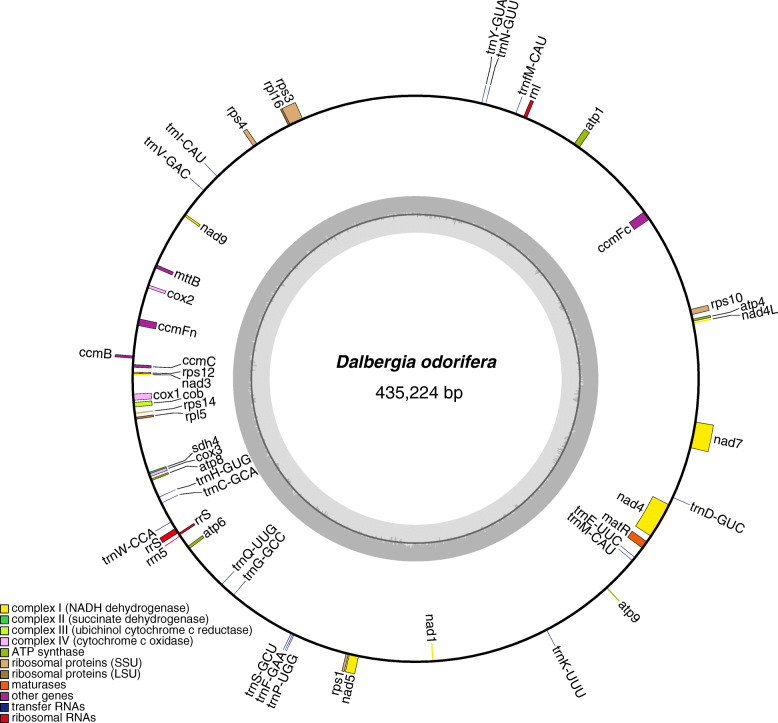
Schematic mitochondrial genome diagram of *D. odorifera*

By employing the sequencing reads from short Illumina reads and long PacBio CLR reads, we successfully assembled the complete mitochondrial genome of *D. odorifera* as a single circular genome. The size of the mitochondrial genome is 435,224 bp (Fig. 1; Table S1), which is similar to most land plants sequenced thus far [10]. The total GC content was 45.1%, which is also like other species in Fabaceae (42.7–45.5%). Based on our comparative analysis, we annotated 54 genes including 33 protein coding genes, 4 rRNA genes, and 17 tRNA genes (Table S1). From the annotated genes, 7 contained introns, with 4 genes (*ccmFc*, *nad5*, *rps10* and *rps3*) containing a single intron and 3 genes (*nad2*, *nad4* and *nad7*) containing more than one intron. Repeat sequences in the mitochondrial genome made up 4.0% of the genome with the longest repeat being 4806 bp in length. As longer repeat sequences can induce structural variation of plant mitochondrial genomes [10, 11, 19], we divided the repeats into four different groups based on length. The groups were: A) less than 20 bp; B) 20–100 bp; C) 101–1000 bp, and D) longer than 1000 bp (A, predicted by MISA; B, C, and D identified by REPuter). The total length of all repeats in each group accounted for 0.13% (585 bp) of the whole genome from group A, 0.82% (3574 bp) from group B, 0.83% (3615 bp) from group C, and 2.2% (9612 bp) from group D. It is also important to note that the presence of longer repeats can induce structural alterations of the mitochondrial genome [19, 20], during different stages of cellular development resulting in arrangements more complex than the simplified circular genome represented here.

### Structure of organellar genomes among Fabaceae

In order to place the features of the *D. odorifera* mitochondrial genome in context, we downloaded all available mitochondrial genomes for which there was a corresponding complete chloroplast genome in NCBI. In total, 20 Fabaceae species were found that met these criteria. The species *E. japonica* (loquat) from Rosaceae was chosen as an outgroup taxon (Table S1).

We compared basic measures of genome size, variability, and content to assess how structural evolution differs across the Fabaceae and between the two organelles. In regard to mitochondrial genome length differences, the smallest genome was from *M. truncatula* as 271,618 bp, and the largest was from *L. trichandra* as 722,009 bp. This amounts were up to a nearly threefold difference in genome size across the Fabaceae, whereas the length difference among the chloroplast genomes for the species sampled is limited with *A. ligulata* possessing the largest (174,233 bp) and *M. truncatula* the smallest (124,033 bp; Table S1). The newly assembled *D. odorifera*, mitochondrial genome was of intermediate length as 435,224 bp compared with the other Fabaceae species sampled in this study. The number of genes among mitochondrial genomes varied (excluding the duplicated protein genes and ORFs) from 25 in *V. angularis* to 39 in *A. mongolicus* (Table S1). The increased number of genes in the *A. mongolicus* mitochondrial genome reflect gene transfers from the chloroplast, rather than mitochondrial duplications. When comparing GC content among the organelles across the Fabaceae, chloroplast genomes range from 34% to 36.8% and mitochondria from 42.7–45.5%. From these results, it is clear that mitochondrial genomes in Fabaceae have greater diversity in structure and content than chloroplasts.

Repeat content for the chloroplast and mitochondrial genomes of 21 species (Table S1) was conducted to assess the diversity among different organelles and lineages (Fig. S1 and S2). The total repeat content in mitochondrial genomes differed from 1.3% (5144 bp) in *V. radiata* to 38.5% (218,282 bp) in *S. tora* compared to chloroplast genomes where the total content ranged from 0.9% (1571 bp) in *A. ligulata* to 8.5% (12,181 bp) in *T. meduseum*. The main difference between the two organelles was in the length of repeats. From the 21 sampled chloroplast genomes (Fig. S1), 19 species possessed many repeats with lengths shorter than 40 bp, with only *A. ligulata* and *T. meduseum* having repeats longer than 40 bp. Among the 21 mitochondrial genomes (Fig. S2), only two species (*M. truncatula* and *V. radiata*) contained any repeats shorter than 40 bp in size with all species containing numerous repeats larger than 50 bp.

**Fig. 2:**
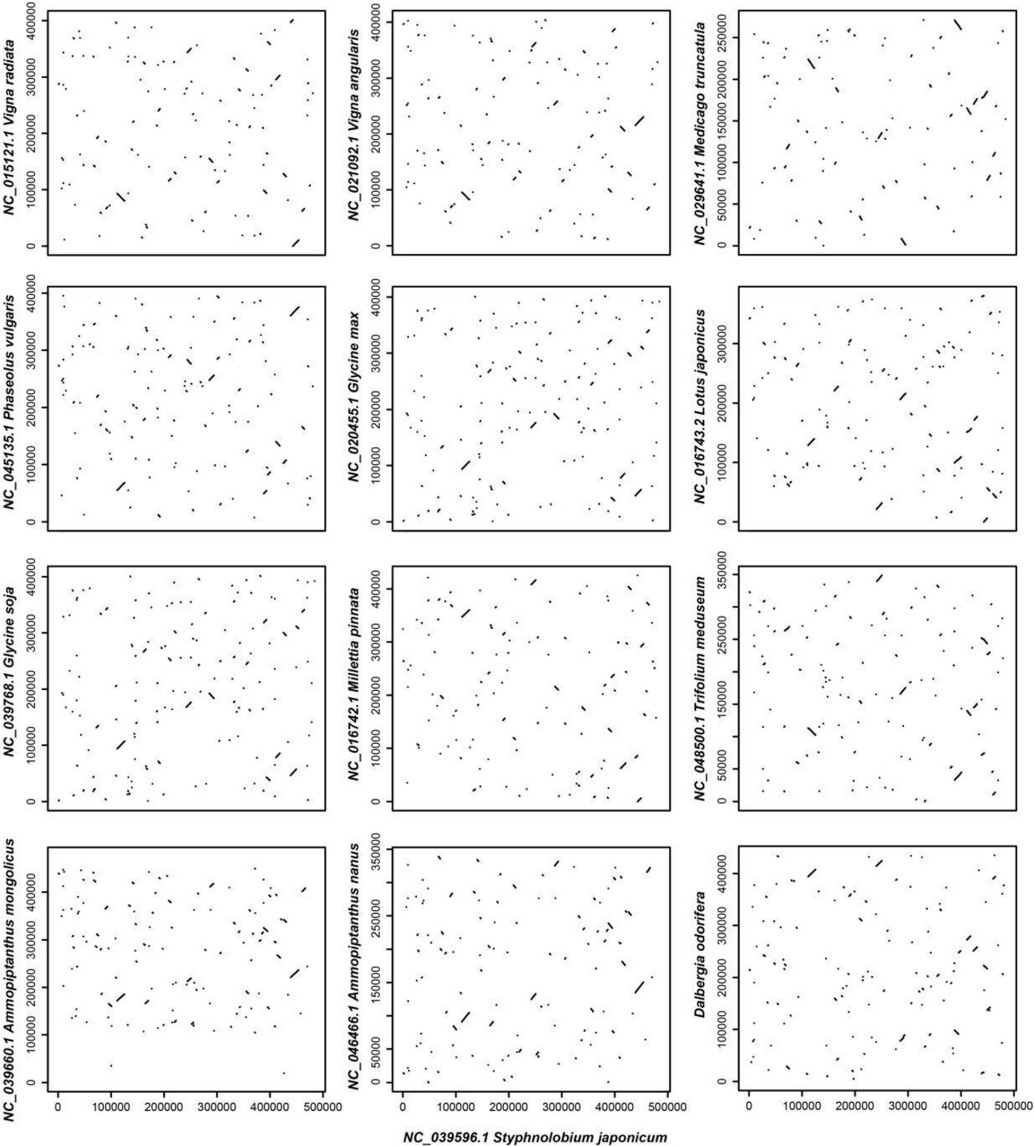
Dot-plot graphs indicating regions of synteny between mitochondrial genomes in Faboideae compared to *S. japonicum* as the reference

Synteny of entire mitochondrial genomes was compared among all Faboideae species in this study to assess the degree of structural rearrangement between different lineages. Because of previous work demonstrating the dynamic nature of plant mitochondrial genome structure [10, 19, 21], including within species [11, 12, 22], this prompted the following analyses to see if any patterns could be discerned. When using *S. japonicum* as a reference genome, the dot-plot analyses showed only short stretches (less than 5Kb) of synteny across all species (Fig. 2). However, when using *Vigna,* or *Ammopiptanthus* as the reference genomes, longer stretches of synteny were found among the interspecific comparisons (Fig. S3 and S4). The pattern of conservation and variability between Faboideae species indicates that mitochondrial genomic synteny decays with time since divergence, while functional genes rearranged within the genome remain internally syntenic and thus highly conserved in protein coding.

### Organellar phylogenetic relationships in Fabaceae

**Fig. 3:**
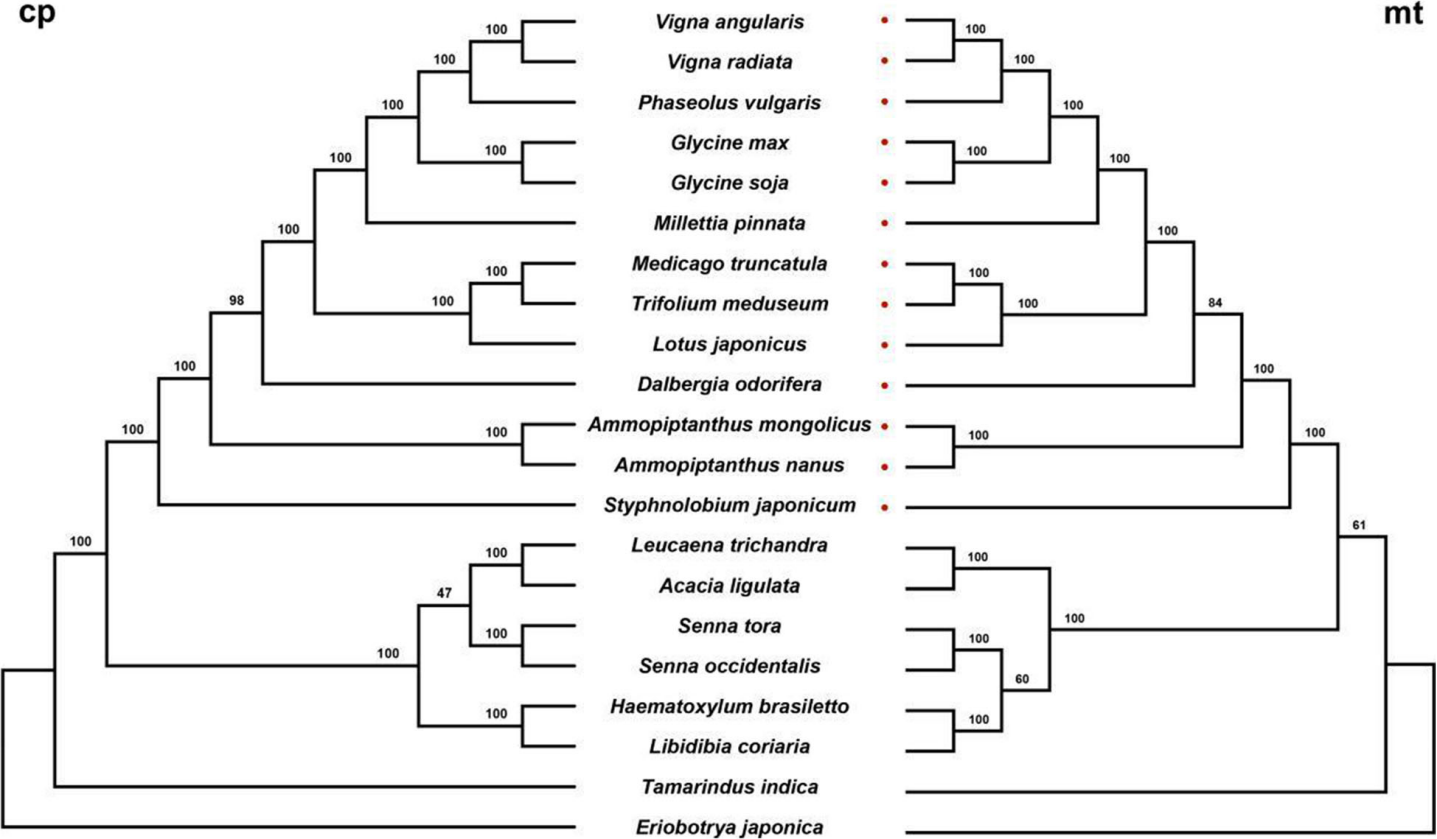
The phylogenic relationships of Fabaceae resolved with chloroplast genes (left) and mitochondrial genes (right). Species with red dots in the mt tree belong to Faboideae

Complete organellar genomes have been used as foundational markers in understanding phylogenetic relationships between species [3, 23, 24]. In our study, we concatenated all shared coding genes from each organelle genome and used these matrices to infer phylogenetic trees from chloroplast and mitochondrial data (Fig. 3). The chloroplast and mitochondrial trees share the same topology, except for branching order in regard to the *Senna* lineage. In the chloroplast tree (Fig. 3), *Leucaena trichandra* + *A. ligulata* are sister to the two *Senna* species, with *Haematoxylum brasiletto* + *L. coriaria* sister to that clade. However, in the mitochondrial tree (Fig. 3), the *Haematoxylum brasiletto* + *L. coriaria* clade is sister to the two *Senna* species. These conflicting topologies were noted by low BS support in both the mitochondrial and chloroplast datasets. Among the remaining branches, topology was identical between the chloroplast and mitochondrial data sets with high support in all cases. When comparing branch lengths between the two trees (Fig. S5), the evolutionary rate among coding genes in the mitochondria appears to be far slower than among chloroplast genes with the exception of *A. mongolicus*. The *A. mongolicus* mitochondrial genome is known to have undergone rapid evolution resulting from chloroplast transfers possibly associated with adaptation to arid environments. However, additional work should be done to confirm these differences are not the result of errors in genome assembly. Other examples of lineage specific accelerated mitochondrial evolution have also been noted from other plant groups [25].

### Sequence transfer between genomes

Characterizing sequence transfer and uptake among the different genomic compartments is essential to understanding intergenomic evolution [8, 9, 14]. By having complete and high-quality assemblies for all three genomes of *D. odorifera*, we were able to evaluate the frequency and pattern of sequence transfer between the organelles and nuclear genome.

1. Length differences of transferred sequences

**Fig. 4:**
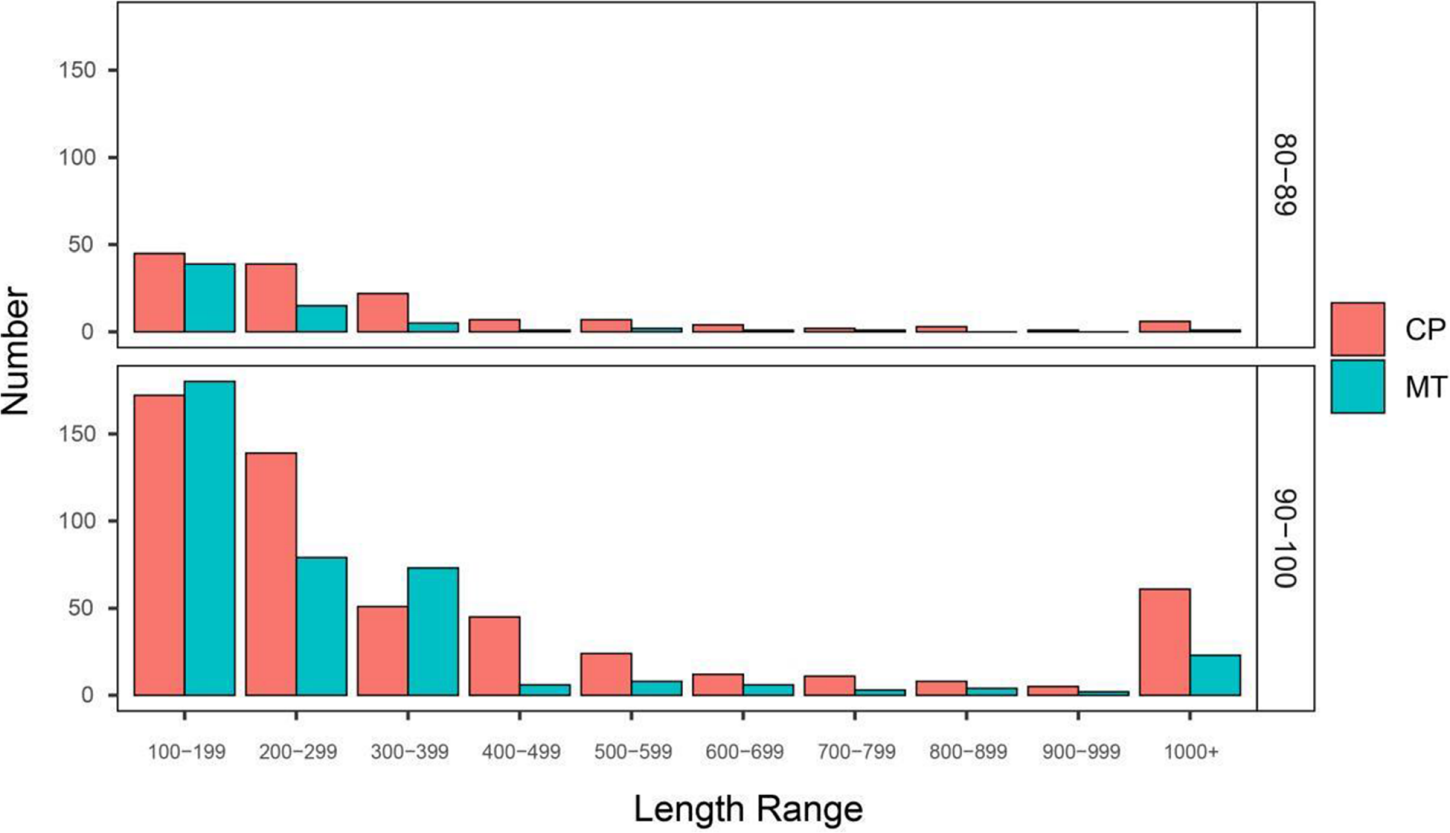
The number and distribution (by size) of transferred sequences from the chloroplast and mitochondrial genome to the nuclear genome

Complete nuclear, mitochondrial, and chloroplast genome assemblies from *D. odorifera* were compared to detect and quantify sequence transfers. The majority (79.5% from chloroplast and 85.5% from mitochondria) of sequence transfers found in this study fell into the 90–100% sequence similarity grouping. In total, the number of sequences transferred to the nuclear genome was greater from the chloroplast than the mitochondria in both the 80–89% and 90–100% sequence similarity groups (Fig. 4). The exception to this was among transferred sequences of 100–200 bp and 300–400 bp categories with 90–100% sequence similarity where more mitochondrial sequences were transferred than chloroplast. The higher abundance of transferred sequences with 90–100% sequence similarity may suggest that the process is continual with sequences being transferred and lost in relatively short periods of time. In addition to quantify the abundance of transfers, the chromosomal location of transfers into the nuclear genome was mapped (Fig. S6). In nine of the ten nuclear chromosomes, chloroplast transfers were more numerous than mitochondrial ones, except in chromosome five where the number of mitochondrial transfers was slightly larger (4.2% vs 4.5%). The total number of sequence transfers into the nuclear genome was similar across all nuclear chromosomes.

2. Features of transferred sequence locations in the nuclear genome

**Fig. 5:**
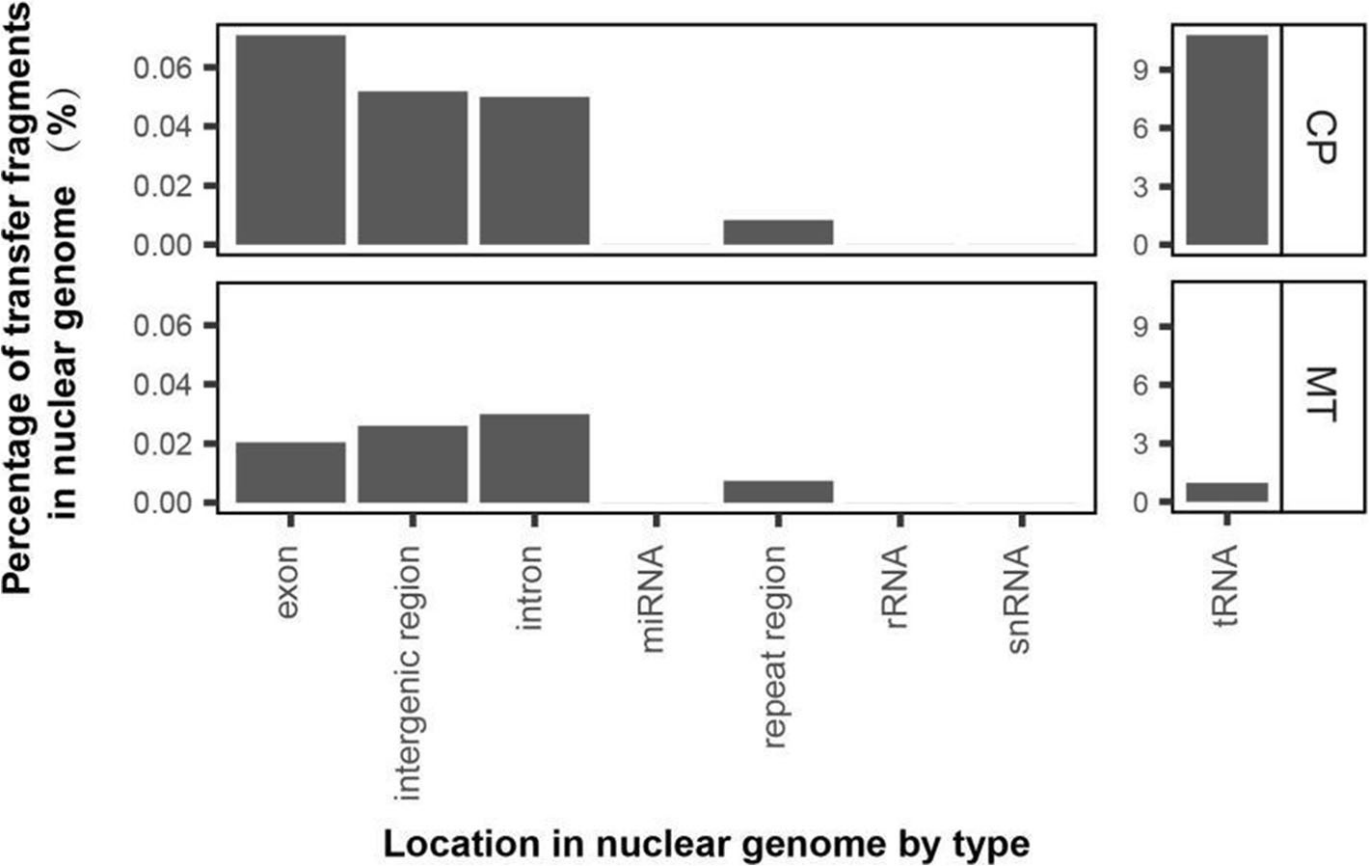
Location type in the nuclear genome of transferred organelle fragments

To further characterize transferred sequences, we identified the locations (by function and/or motif type) in which the transfers were found in the nuclear genome (Fig. 5). Based on the sequence content of the nuclear genome and annotations thereof, the transfer locations were divided into eight categories including exon, intron, rRNA, tRNA, snRNA, miRNA, intergenic DNA, and repetitive regions. Reflecting the results from the length of transfers analysis, all transfer location types were predominantly occupied by sequences of chloroplast origin. The most common location for transfers from both the chloroplast and mitochondria was in tRNA, making up 10.8 and 1.0% of nuclear tRNA respectively. The second most abundant target for transferred chloroplast fragments was into nuclear exons (0.07%) and for mitochondrial sequences, introns (0.02%). Transfer sequence location types were mapped onto the nuclear chromosomes to confirm if any patterns were present. For the intronic regions, chromosome 5 contained the highest number of fragments from the mitochondrial genome. For most tRNA regions, we found chloroplast fragments at each chromosome, but mitochondrial fragments were only present in chromosomes 2–4 and 10 (Fig. S7). In addition, nuclear GC content variation flanking the insertion sites was quantified to assess whether any consistent patterns were associated with insertion. For the transferred chloroplast fragments, there was very little difference in GC content at any of the insertion sites irrespective of the size of the insert. For the transferred mitochondrial fragments, the 100–500 bp groups had no difference in GC content. However, for the groups including 500–1000+, the 3′ side had a higher GC content than the 5′ side for the first 50-100 bp from the insert (Fig. S8 and S9). Beyond the 50–100 flanking regions GC content was essentially equivalent.

## Discussion

### The assembly of plant mitochondrial genomes

Plant mitochondrial genomes have undergone dramatic and rapid structural changes since the initial endosymbiosis event [9, 19]. Due to this mode of evolution, mitochondrial genome composition is complex, making conventional modes of sequencing and assembly less effective [26–28]. The simple circular model of genome structure that applies to most animal species is inadequate when trying to understand plant mitochondria that can have multiple circulars, branched, linear, or mixed forms of genomic structure [19]. In this study, to complet the mitochondrial genome of *D. odorifera*, we employed short Illumina reads combined with long PacBio CLR reads to overcome issues with assembling complex genomes of this type. The circular assembled mitochondrial genome was 435,224 bp in length with 33 annotated protein-coding genes. Circular arrangements appear to be the common form among mitochondrial genomes assembled thus far in Fabaceae (Table S1). That said, during assembly of the *D. odorifera* mitochondrial genome, several sites were found that could form branching or sub-circular structures (Data not shown). This suggests that the *D. odorifera* mitochondrial genome may take alternative forms during different stages of development or in different tissue types as has been found in other plant species [12, 29]. More work is needed to understand this phenomenon among plant mitochondrial genomes, but with long read single cell sequencing, elucidating this process is now more tractable. To these ends, the first complete mitochondrial genome in *Dalbergia* will provide a useful reference for follow-up work in understanding the function and structuring of plant mitochondria.

### The evolution of mitochondrial genomes in *Fabaceae*

Plant mitochondria are known to evolve rapidly even within a species, with differences between individuals varying by thousands of bases outside of functional genes, resulting in a broad distribution of genome sizes [12, 19, 21, 22]. Furthermore, heteroplasmy, large scale genome recombination, and gene chimerism are all known to occur among mitochondrial genomes at the species and individual level and are associated with, but may not be responsible for, certain phenotypes [22, 30]. When comparisons are made across more distant lineages, the differences in genome size and structure are even more pronounced yet functional genes remain conserved [11, 31, 32].

While mitochondrial genomes for numerous crop species have been sequenced and studied owing to their economic importance, far fewer mitochondrial genomes from large long-lived species such as *D. odorifera* have been sequenced. Given that the Fabaceae contain numerous important crop species as well as large long-lived tree species and numerous other forms, this is a superb model lineage to study the evolution of organelle genomes and the relation of these evolutionary changes to physiological and phylogenetic processes such as speciation. The mitochondrial genome of *D. odorifera* is the first species within the tribe Dalbergieae (Faboideae), for which all three genomic compartments have been sequenced. From this we were able compare the Dalbergieae lineage organelles to organelles from other important lineages in Faboideae and Fabaceae. For the chloroplast genome, structural variation was limited in the Faboideae clade just as the 50 Kb inversion in the large single copy region of the chloroplast (Fig. S10). Among chloroplasts, variation in gene content was also limited reflecting the conservation of function found in other lineages [11, 33].

In addition to the ML method employed in IQTREE, Bayesian analyses was also conducted in MrBayes to ensure that the gene-based matrix was robust for use in phylogenetic reconstruction. Gene partition analyses was also conducted in IQTREE and MrBayes to assess any effects from differential rates of gene evolution on tree topology. No differences in topology were noted but some differences in branch length and support were observed (Figs. S11, S12, S13, S14). From the phylogenetic comparison of the chloroplast and mitochondrial genes in the Fabaceae (Fig. 3 and Fig. S5), tree branch lengths are longer among chloroplast genes than in mitochondrial genes. The exception to this is the long branch of *A. mongolicus* which might be the result of numerous chloroplast transfers to the mitochondria. Interestingly *A. mongolicus* is thought to have undergone accelerated evolution since the Tertiary as a response to rapid desertification in northwestern China. Such drastic changes to the mitochondrial genome through chloroplast transfers may have been part of this rapid adaptation. The slow rate of mitochondrial gene evolution is in stark contrast to the rapid evolution detected in all other sections of the mitochondrial genome. In fact, the turnover in genome structure is so rapid among mitochondrial genomes, synteny beyond 5 Kb sections is nonexistent when comparing lineages above the generic level in Fabaceae (Fig. 2). Such differences in rate of sequence and structure evolution are thought to be a result of mitochondrial break-induced repair mechanisms. In regard to phylogenetic topology, the chloroplast and mitochondrial trees are very similar, as is expected among uniparentally non-recombinant genomes (Fig. 3). However, several instances of phylogenetic discordance were noted which may be the result of several factors including limited sampling and differences in evolutionary rates and/or incomplete lineage sorting after a biparental organellar inheritance event (which has been documented in Faboideae) [34, 35]. To better understand the evolution of mitochondrial genomes, a greater density of sampling from the population to family level needs to be conducted. Given the broad diversity of body plans, lifecycles, number of species, and the progress thus far in sequencing, the Fabaceae is an excellent lineage to test the numerous hypotheses regarding genome evolution in plants. Sequencing and assembling the first complete mitochondrial genome in the Dalbergieae is just the first step to understand the variation of mitochondrial genome in this family.

### Intergenomic sequence transfers

Sequence transfer among genomic compartments appears to be a salient and continuously evolving trait of the endosymbiotic process, given the number of detectable sequences found between the different genomes in plants [9, 36]. Many studies have been conducted to help explain the mechanisms of sequence transference, such as incidental incorporation caused by the illegitimate repair of double-stranded breaks [36] or as part of stress or other responses to induce functional genetic diversity in the receiving genome [37]. No matter what kinds of the mechanisms, more work is needed to simply document the diversity of transfers across plants, to find patterns in the location, abundance, timing, and biases of transfers which may still need to generate appropriate hypotheses to test.

For the first-time, total sequence transfer from all genomic compartments was assessed in the Dalbergieae species *D. odorifera*. From our analyses, it was clear that chloroplast transfers were more common than mitochondrial as about 92% (120,221 bp) of the chloroplast genome was present in the nuclear genome, while only 26% (113,888 bp) was found from the mitochondria. That said the total length contributed from each genome was similar in the nuclear genome. A bias in chloroplast transfers has been found in other species, where this has been studied and might be related to the fact that chloroplasts exist in higher abundance within a cell than mitochondria. Compared to the 83% of chloroplast transfer to the nuclear genome in *Oryza* and 16% in *Arabidopsis* [38], the 92% chloroplast transfer found in *D. odorifera* is high. However other instances of abundant chloroplast sequence transfer were found in our study such as in the *A mongolicus* mitochondria, suggesting that high rates of chloroplast sequence transfer may be especially common in the *Fabaceae*. More work on what drives these transfers and subsequent retention and purging in the receiving genome is needed to deduce why such different transfer rates are found among the plant species studied thus far.

## Conclusions

 Though the nuclear and chloroplast genomes of *D. odorifera* have already been published [3], the addition of the complete mitochondrial genome allowed for comprehensive comparisons to be made across the genomic compartments within an individual and to other lineages allowing for a broader prospective in studying evolutionary changes. The use of long-read and short-read sequencing makes possible to accurately assemble the plant mitochondrial genomes, which was due to the limited synteny between even closely related lineages. In the future, this approach can be applied more broadly to sequence mitochondrial genomes from different tissues, populations, and species to decipher how structural and function evolution have shaped the highly diverse plant mitochondrial genomes. Such knowledge can be applied to predictive breeding studies, organellar transplantation, and a range of different gene editing constructs as well as targets. The Fabaceae and *Dalbergia* are a tractable group of plants to study further, given their diversity and applicability to numerous humans uses as well as an often-essential role in the ecosystems in which they exist. The very diversity of the Fabaceae may have come about due to the ability of certain lineages to adapt quickly during times of rapid change, which in turn might be mediated by intergenomic sequence transfer*.*

## Methods

### Genome assembly and annotation

The genome sequencing data from Illumina short-reads and PacBio CLR long sequencing reads [3] of *D. odorifera* were downloaded from NCBI (PRJNA613774) for mitochondrial genome assembly. Considering the high copy number of mitochondrial sequences within a cell, approximately 6 Gb of Illumina reads were extracted randomly from a complete genome sequencing effort to generate a draft mitochondrial genome using Spades v3.14.0 software [39] with default parameters. The published mitochondrial genomes in NCBI were used to search for mitochondrial sequences from the draft mitochondrial genome assembled in Spades v3.14.0 from illumina data. Then the extracted mitochondrial sequences were used to select a total of 169 Mb of CLR mitochondrial reads, which were further corrected with all CLR reads using minimap2 v2.17-r941 [40] and Racon v1.4.13 [41]. The corrected reads were assembled by Flye v2.7-b1585 [42] to obtain the final mitochondrial genome. The PacBio assembly was ‘polished’ using Illumina reads in Pilon v1.23 [43]. The complete assembled circular genome was then annotated to delimit the locations of functional genes.

To obtain accurate annotations for the *D. odorifera* mitochondrial genome, Geseq (https://chlorobox.mpimp-golm.mpg.de/geseq.html) [44] was used with the species *S. japonicum* (NC_039596.1), *M. truncatula* (NC_029641.1), *Lotus japonicus* (NC_016743.2), *V. radiata* (NC_015121.1), *V. angularis* (NC_021092.1), *G. max* (NC_020455.1), *G. soja* (NC_039768.1), *A. mongolicus* (NC_039660.1), *S. occidentalis* (NC_038221.1), *S. tora* (NC_038053.1), *Leucaena trichandra* (NC_039738.1) and *A. ligulata* (NC_040998.1) as references. The Public MITOFY Analysis Web Server (https://vcru.wisc.edu/cgi-bin/mitofy/mitofy.cgi) [45] was used to verify the annotation results. Finally, the visualization of genome structure was implemented using the Draw Organelle Genome Maps online software (OGDRAW v1.3.1, https://chlorobox.mpimp-golm.mpg.de/OGDraw.html) [46], and the assembly and annotation files were submitted to NCBI under the accession number MW441235.

### Phylogenetic analysis and comparisons of synteny

To analyze the phylogenetic relationships among Fabaceae, the chloroplast, and mitochondrial genomes of *D. odorifera* and 19 other Fabaceae were used, with *E. japonica* (Rosaceae) as an outgroup (Table S1). The CDS sequences of 77 chloroplast protein-coding genes and 32 mitochondrial protein-coding genes were extracted into two separate files (one for mitochondria and one for chloroplast), concatenated, and aligned using MAFFT v7.464 [47, 48], with poorly aligned sections trimmed with TrimAL v1.4 [49] to obtain two organelle datasets. These datasets were then used to conduct two separate phylogenetic analysis using IQ-TREE v2.0 [50, 51] with 1000 ultrafast bootstrap replicates to assess branch support based on the auto-selected best-fit model ‘TVM + F + R4’, with FigTree v1.4.3 (http://tree.bio.ed.ac.uk/software/figtree) used for tree visualization. Trees were run with separate gene partition matrices to see how tree topology, branch length, and branch support were affected with the parameters ‘-p -m’ in IQTREE. PartitionFinder v2.1.1 [52] and MrBayes v3.2.7a [53] were also used to generate the Bayesian phylogenetic tree in order to assess the robustness of the gene-based matrix across methods.

The synteny among Faboideae mitochondrial genomes was verified with sequence similarity. The mitochondrial genomes of 12 Faboideae species were compared with the *S. japonicum* using BLASTn [54] with the filter parameter ‘percent identity more than 80 and alignment length more than 100’. The synteny results were visualized using the plot function in R v3.3.1.

### Fragment transfer analysis

Based on the assembly and annotation files of the *D. odorifera* nuclear genome [3], BLASTn software [54] was used to identify transfer events from organelle to nuclear genomes. The results were grouped into two datasets, one of which had 80–89% identity scores, and another with 90–100% identity scores, which were used to represent different transfer timing, due to newer transfers usually having higher identity scores. These two datasets were further split with different cut-off of alignment lengths of 100–500 bp, 500–1000 bp and more than 1000 bp. In total six datasets were generated for each organelle genome. Visualization of these results was implemented with Circos v0.69–8 [55].

The *D. odorifera* nuclear genome organelle fragments were annotated as transferred sequences using BEDtools v2.27.0 [56] and subsequently used to assess transfer location distribution patterns in the nuclear genome. The 5′ and 3′ flanking regions of transferred fragments in the chromosome were extracted to calculate the GC content. The ggplot2 v3.3.3 package [57] in R was used to graphically present the results.

### Repeat analysis of organelle genome

Four repeat types were assessed across all 21 species used in this study for both organelle genomes. The repeat types, F (forward), P (palindrome), R (reverse), and C (complement) were identified using REPuter [58] with default settings. The MISA software [59, 60] was used to identify simple sequence repeats (SSRs) in *D. odorifera*, with 10, 6, 5, 5, 5, and 5 repeat units set as minimum thresholds for mono-, di-, tri-, tetra-, penta-, and hexa-motif microsatellite identification respectively.

## Supplementary Information

**Additional file 1: Table S1**. The sampled species from Fabaceae and their organelle genome features.

**Additional file 2: Fig. S1.** Repeats found in the chloroplast genome of *D. odorifera.* Repeats detected are: F (forward direct match repeats), R (reverse match repeats), C (complement match repeats), P (palindromic match repeats). **Fig. S2.** Repeats found in the mitochondrial genome of *D. odorifera.* Repeats detected are: F (forward direct match repeats), R (reverse match repeats), C (complement match repeats), P (palindromic match repeats). **Fig. S3.** Dot-plot graphs indicating collinearity of mitochondrial genomes in Faboideae as compared with *V. radiata* for reference. **Fig. S4.** Dot-plot graphs indicating collinearity of mitochondrial genomes in Faboideae as compared with *Ammopiptanthus nanus* for reference. **Fig. S5.** The phylogenic relationships of Fabaceae organelles as inferred for chloroplast genes (left) and mitochondrial genes (right) with proportional branch lengths. **Fig. S6.** The percent of transferred sequence in each nuclear chromosome. **Fig. S7.** The frequency of organelle DNA transferred in each nuclear chromosome. **Fig. S8.** The GC content of nuclear genome flanking sequences adjacent to inserted chloroplast fragments. **Fig. S9.** The GC content of nuclear genome flanking sequences adjacent to inserted mitochondrial fragments. **Fig. S10.** Dot-plot graphs indicating collinearity of chloroplast genomes in Faboideae as compared with *S. japonicum* for reference. **Fig. S11**. The bayes phylogenic tree of Fabaceae by using the chloroplast genes. **Fig. S12**. The bayes phylogenic tree of Fabaceae by using the mitchondrial genes. **Fig. S13**. The phylogenic tree of Fabaceae chloroplast genes by using partition method. **Fig. S14**. The phylogenic tree of Fabaceae mitchondrial genes by using partition method.

## Data Availability

All sequencing data have been submitted to NCBI and are available via accession number MW441235. The other datasets used in this study are available in the NCBI. *Acacia ligulata* chloroplast genome, accession number: NC_040998.1, DOI: https://www.ncbi.nlm.nih.gov/nuccore/NC_040998.1/;*A. mongolicus* chloroplast genome, accession number: NC_039660.1, DOI: https://www.ncbi.nlm.nih.gov/nuccore/NC_039660.1/;*A. nanus* chloroplast genome, accession number: NC_046466.1, DOI: https://www.ncbi.nlm.nih.gov/nuccore/NC_046466.1/;*D. odorifera* chloroplast genome, accession number: MW441235, DOI: https://www.ncbi.nlm.nih.gov/nuccore/MW441235/;*E. japonica* chloroplast genome, accession number: NC_045228.1, DOI: https://www.ncbi.nlm.nih.gov/nuccore/NC_045228.1/;*G. max* chloroplast genome, accession number: NC_020455.1, DOI: https://www.ncbi.nlm.nih.gov/nuccore/NC_020455.1/;*G. soja* chloroplast genome, accession number: NC_039768.1, DOI: https://www.ncbi.nlm.nih.gov/nuccore/NC_039768.1/;*Haematoxylum brasiletto* chloroplast genome, accession number: NC_045040.1, DOI: https://www.ncbi.nlm.nih.gov/nuccore/NC_045040.1/;*Leucaena trichandra* chloroplast genome, accession number: NC_039738.1, DOI: https://www.ncbi.nlm.nih.gov/nuccore/NC_039738.1/;*L. coriaria* chloroplast genome, accession number: NC_045039.1, DOI: https://www.ncbi.nlm.nih.gov/nuccore/NC_045039.1/;*Lotus japonicus* chloroplast genome, accession number: NC_016743.2, DOI: https://www.ncbi.nlm.nih.gov/nuccore/NC_016743.2/;*M. truncatula* chloroplast genome, accession number: NC_029641.1, DOI: https://www.ncbi.nlm.nih.gov/nuccore/NC_029641.1/;*M. pinnata* chloroplast genome, accession number: NC_016742.1, DOI: https://www.ncbi.nlm.nih.gov/nuccore/NC_016742.1/;*P. vulgaris* chloroplast genome, accession number: NC_045135.1, DOI: https://www.ncbi.nlm.nih.gov/nuccore/NC_045135.1/;*S. occidentalis* chloroplast genome, accession number: NC_038221.1, DOI: https://www.ncbi.nlm.nih.gov/nuccore/NC_038221.1/;*S. tora* chloroplast genome, accession number: NC_038053.1, DOI: https://www.ncbi.nlm.nih.gov/nuccore/NC_038053.1/;*S. japonicum* chloroplast genome, accession number: NC_039596.1, DOI: https://www.ncbi.nlm.nih.gov/nuccore/NC_039596.1/;*T. indica* chloroplast genome, accession number: NC_045038.1, DOI: https://www.ncbi.nlm.nih.gov/nuccore/NC_045038.1/;*T. meduseum* chloroplast genome, accession number: NC_048500.1, DOI: https://www.ncbi.nlm.nih.gov/nuccore/NC_048500.1/;*V. angularis* chloroplast genome, accession number: NC_021092.1, DOI: https://www.ncbi.nlm.nih.gov/nuccore/NC_021092.1/;*V. radiata* chloroplast genome, accession number: NC_015121.1, DOI: https://www.ncbi.nlm.nih.gov/nuccore/NC_015121.1/; *A. ligulata* mitochondrial genome, accession number: NC_026134.2, DOI: https://www.ncbi.nlm.nih.gov/nuccore/NC_026134.2/;*A. mongolicus* mitochondrial genome, accession number: NC_034742.1, DOI: https://www.ncbi.nlm.nih.gov/nuccore/NC_034742.1/;*A. nanus* mitochondrial genome, accession number: NC_034743.1, DOI: https://www.ncbi.nlm.nih.gov/nuccore/NC_034743.1/;*D. odorifera* mitochondrial genome, accession number: MT644131, DOI: https://www.ncbi.nlm.nih.gov/nuccore/MT644131/;*E. japonica* mitochondrial genome, accession number: NC_034639.1, DOI: https://www.ncbi.nlm.nih.gov/nuccore/NC_034639.1/;*G. max* mitochondrial genome, accession number: NC_007942.1, DOI: https://www.ncbi.nlm.nih.gov/nuccore/NC_007942.1/;*G. soja* mitochondrial genome, accession number: NC_022868.1, DOI: https://www.ncbi.nlm.nih.gov/nuccore/NC_022868.1/;*H. brasiletto* mitochondrial genome, accession number: NC_026679.1, DOI: https://www.ncbi.nlm.nih.gov/nuccore/NC_026679.1/;*L. trichandra* mitochondrial genome, accession number: NC_028733.1, DOI: https://www.ncbi.nlm.nih.gov/nuccore/NC_028733.1/;*L. coriaria* mitochondrial genome, accession number: NC_026677.1, DOI: https://www.ncbi.nlm.nih.gov/nuccore/NC_026677.1/;*L. japonicus* mitochondrial genome, accession number: NC_002694.1, DOI: https://www.ncbi.nlm.nih.gov/nuccore/NC_002694.1/;*M. truncatula* mitochondrial genome, accession number: NC_003119.8, DOI: https://www.ncbi.nlm.nih.gov/nuccore/NC_003119.8/;*M. pinnata* mitochondrial genome, accession number: NC_016708.2, DOI: https://www.ncbi.nlm.nih.gov/nuccore/NC_016708.2/;*P. vulgaris* mitochondrial genome, accession number: NC_009259.1, DOI: https://www.ncbi.nlm.nih.gov/nuccore/NC_009259.1/;*S. occidentalis* mitochondrial genome, accession number: NC_038222.1, DOI: https://www.ncbi.nlm.nih.gov/nuccore/NC_038222.1/;*S. tora* mitochondrial genome, accession number: NC_030193.1, DOI: https://www.ncbi.nlm.nih.gov/nuccore/NC_030193.1/;*S. japonicum* mitochondrial genome, accession number: NC_045071.1, DOI: https://www.ncbi.nlm.nih.gov/nuccore/NC_045071.1/;*T. indica* mitochondrial genome, accession number: NC_026685.1, DOI: https://www.ncbi.nlm.nih.gov/nuccore/NC_026685.1/;*T. meduseum* mitochondrial genome, accession number: NC_024166.1, DOI: https://www.ncbi.nlm.nih.gov/nuccore/NC_024166.1/;*V. angularis* mitochondrial genome, accession number: NC_021091.1, DOI: https://www.ncbi.nlm.nih.gov/nuccore/NC_021091.1/;*V. radiata* mitochondrial genome, accession number: NC_013843.1, DOI: https://www.ncbi.nlm.nih.gov/nuccore/NC_013843.1/.
