# Supplementary material for: A complete mitochondrial genome for fragrant Chinese rosewood (Dalbergia odorifera, Fabaceae) with comparative analyses of genome structure and intergenomic sequence transfers
Source: BMC Genomics. 2021 Sep 18;22:672. doi: 10.1186/s12864-021-07967-7 (PMC8449883; doi:10.1186/s12864-021-07967-7)

**Supplementary Information**

**Figure S1.** Repeats found in the chloroplast genome of *D. odorifera.* Repeats detected are: F (forward direct match repeats), R (reverse match repeats), C (complement match repeats), P (palindromic match repeats).


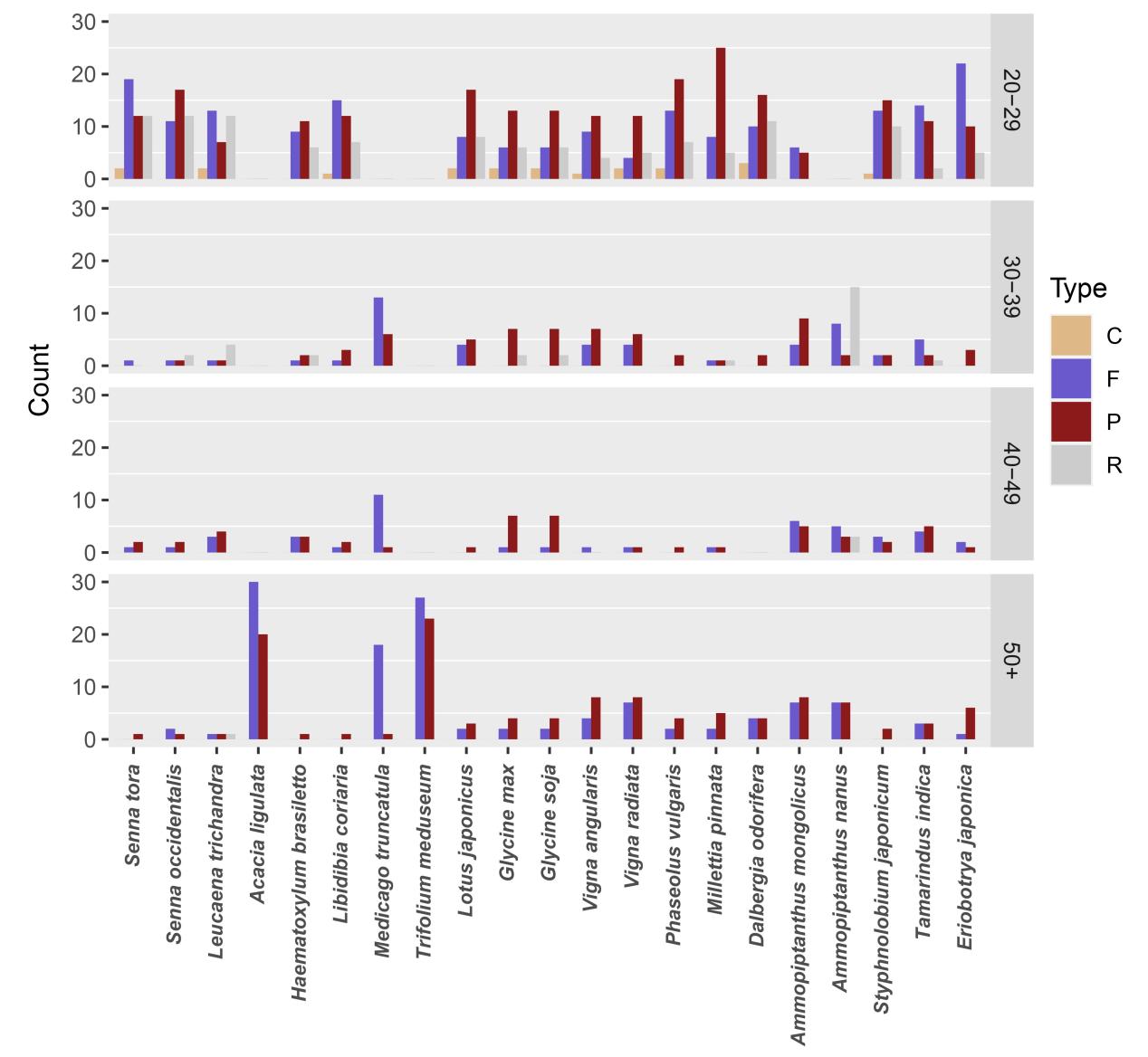


**Figure S2.** Repeats found in the mitochondrial genome of *D. odorifera.* Repeats detected are: F (forward direct match repeats), R (reverse match repeats), C (complement match repeats), P (palindromic match repeats).


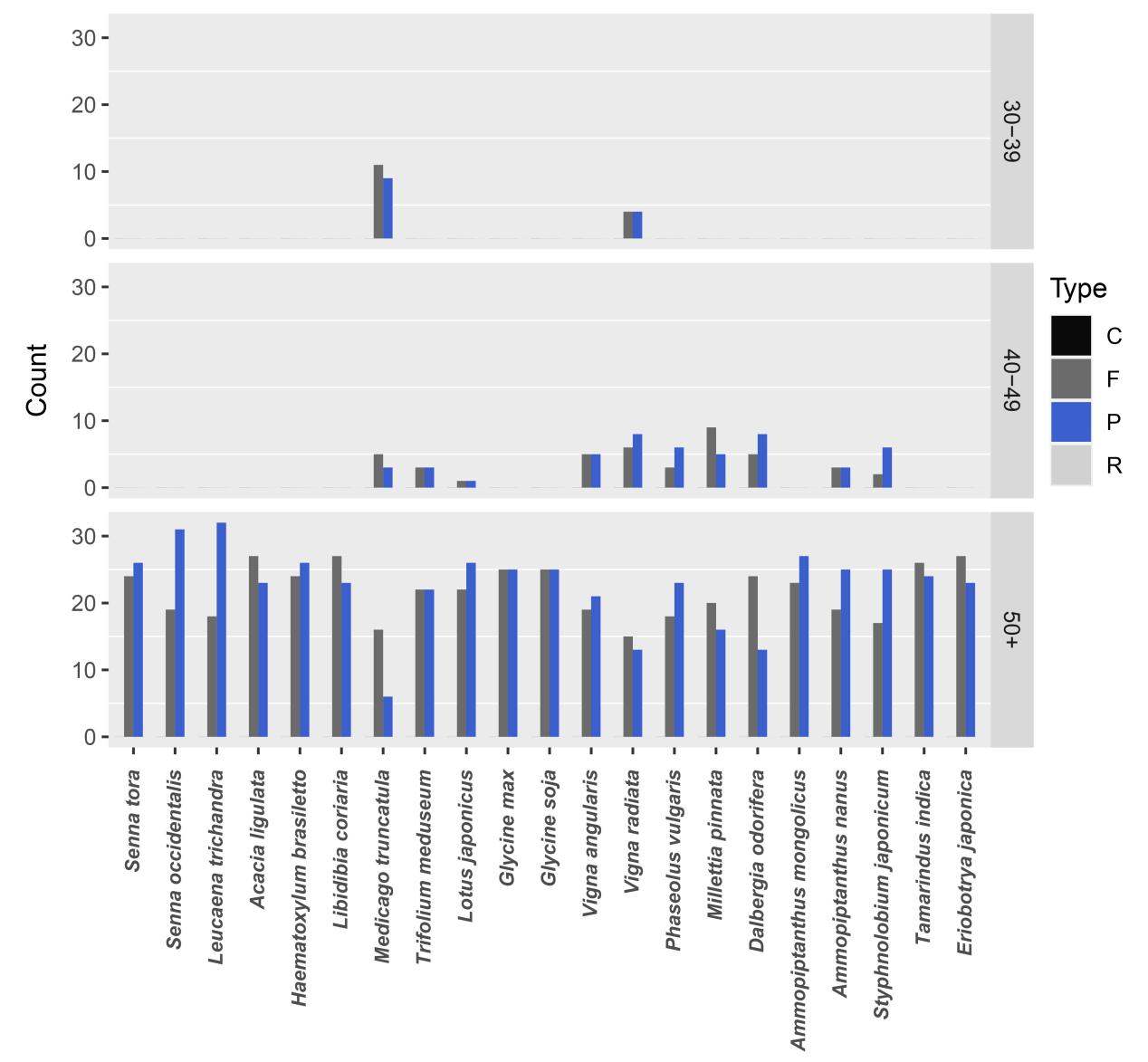


**Figure S3.** Dot-plot graphs indicating collinearity of mitochondrial genomes in Faboideae as compared with *Vigna radiata* for reference.


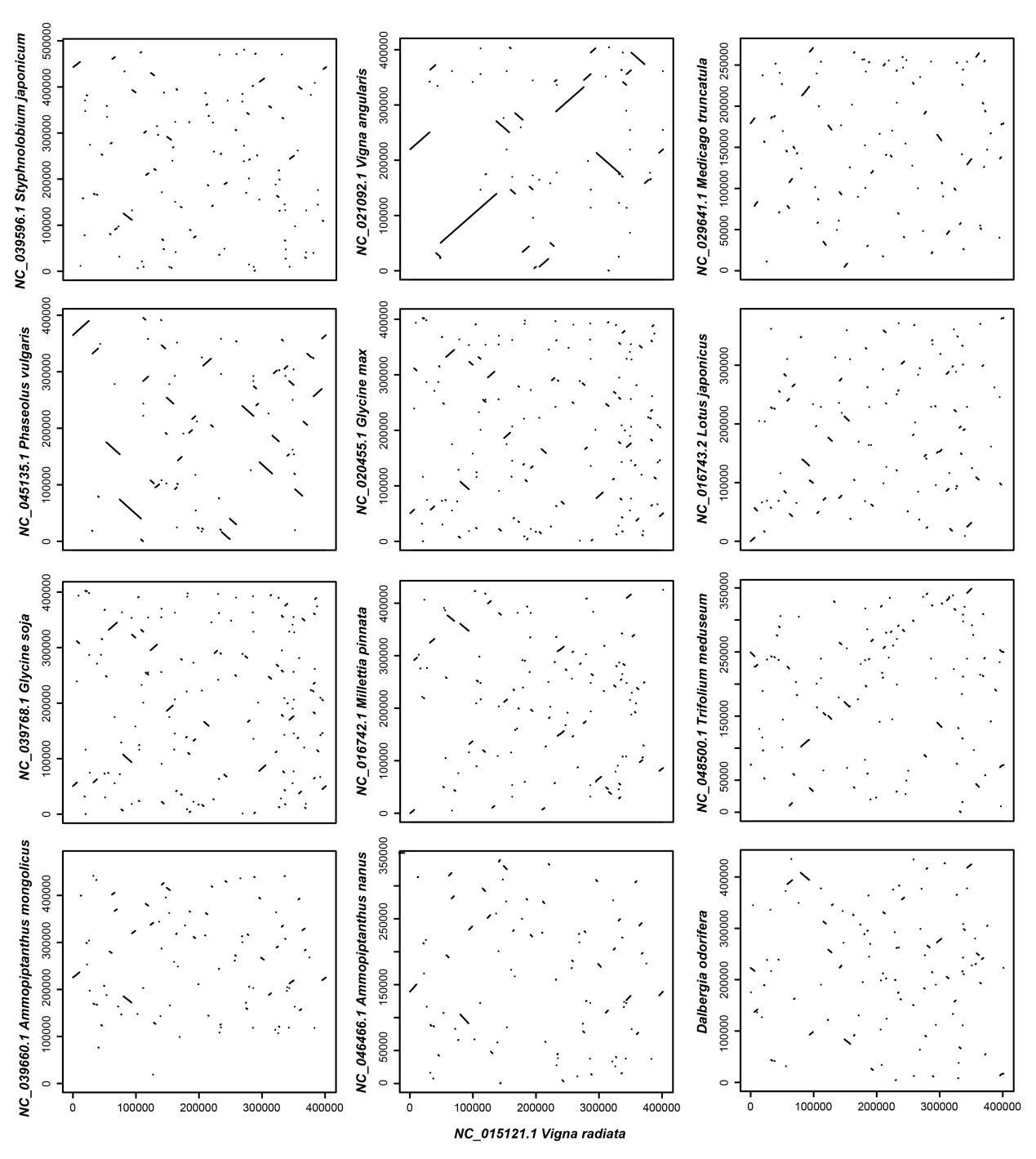


**Figure S4.** Dot-plot graphs indicating collinearity of mitochondrial genomes in Faboideae as compared with *Ammopiptanthus nanus* for reference.


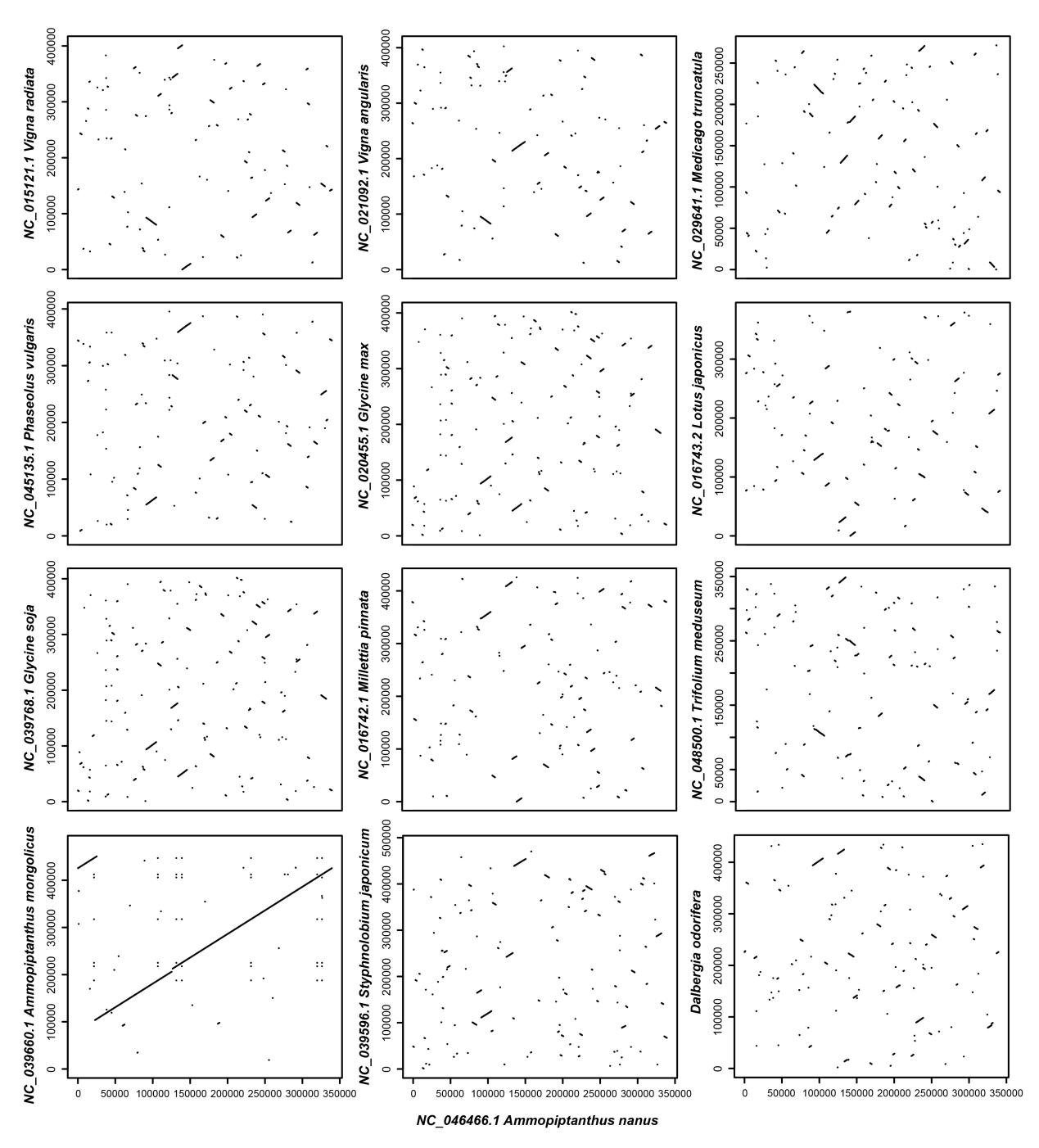


**Figure S5.** The phylogenic relationships of Fabaceae organelles as inferred for chloroplast genes (left) and mitochondrial genes (right) with proportional branch lengths.


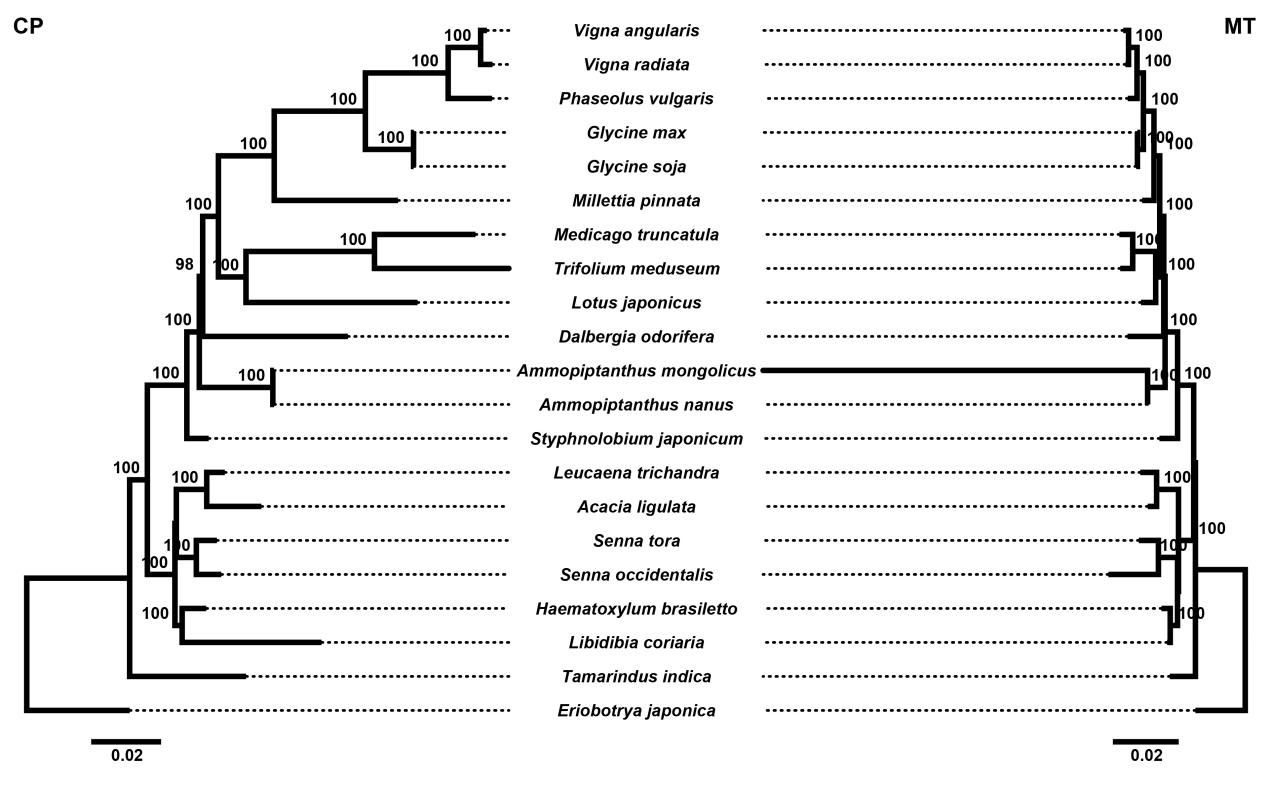


**Figure S6.** The percent of transferred sequence in each nuclear chromosome.


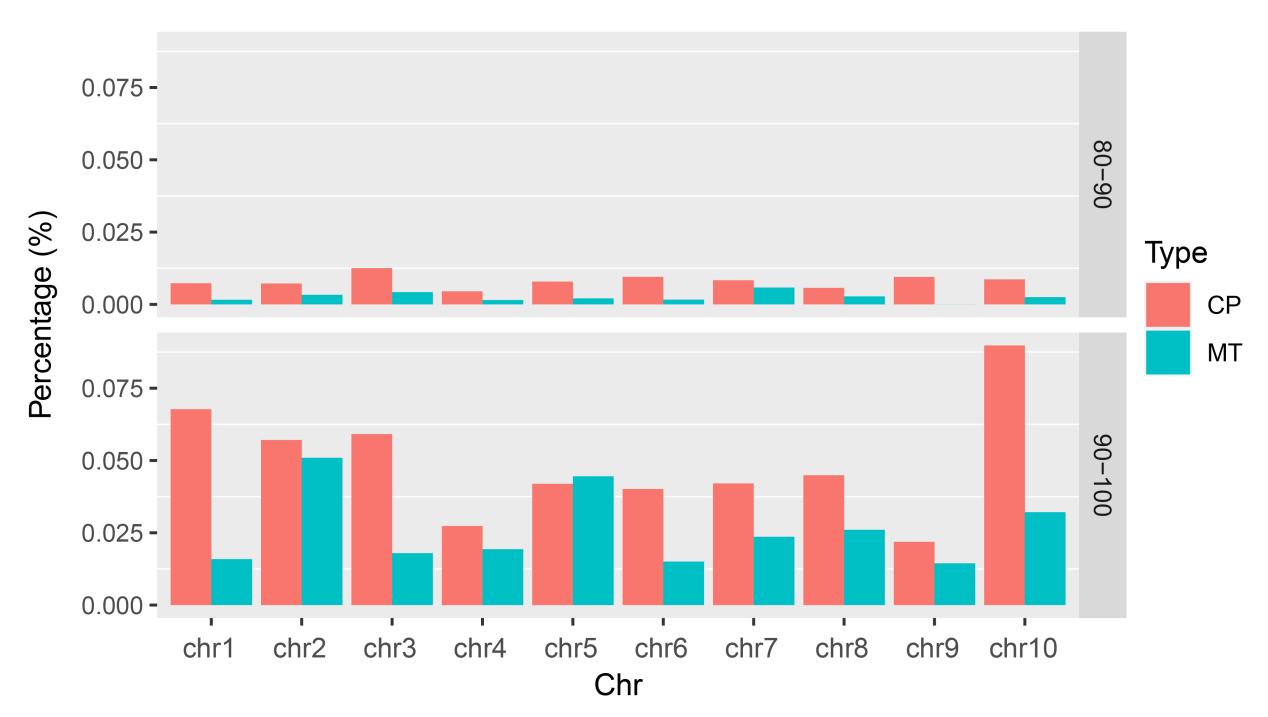


**Figure S7.** The frequency of organelle DNA transferred in each nuclear chromosome.


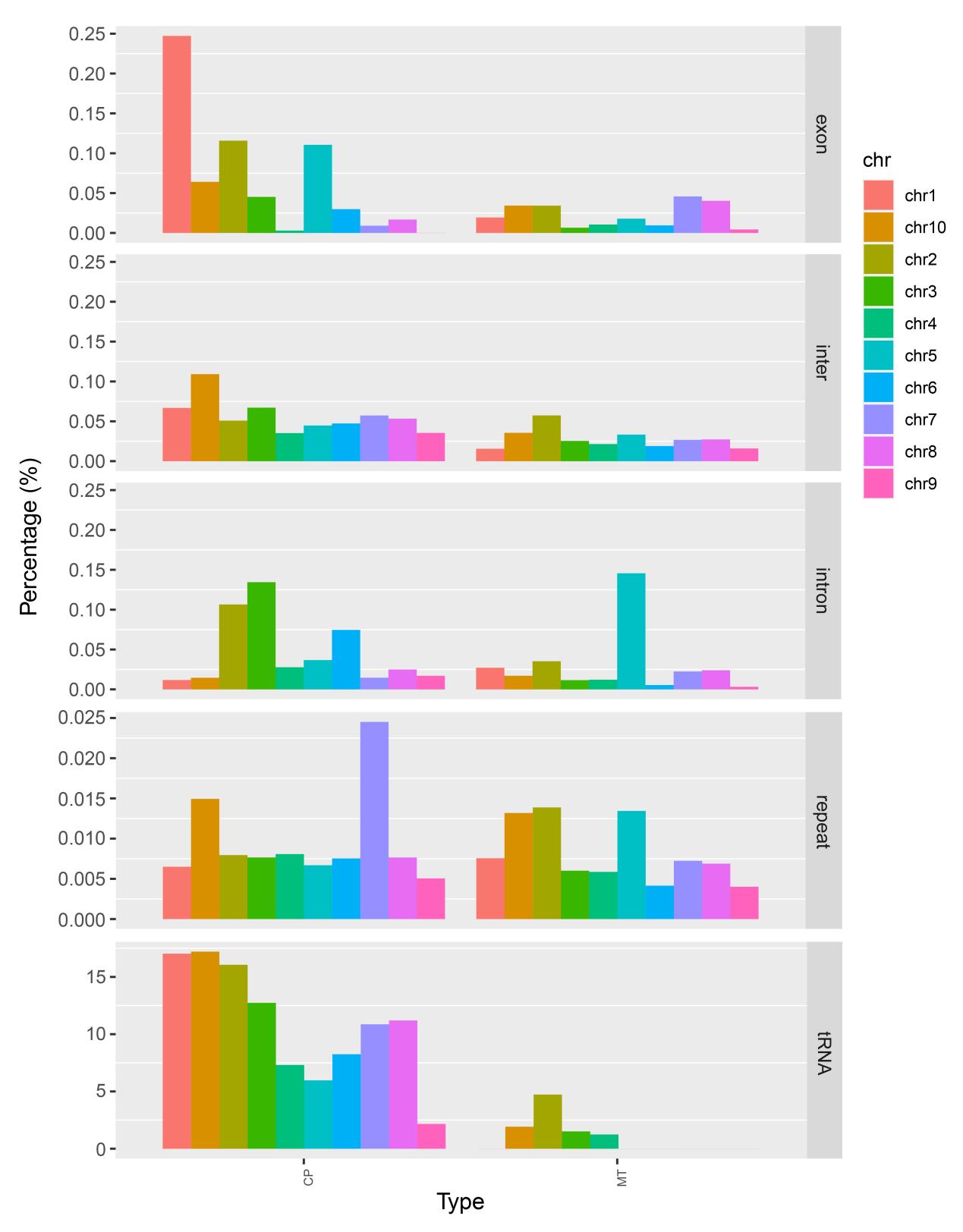


**Figure S8.** The GC content of nuclear genome flanking sequences adjacent to inserted chloroplast fragments.


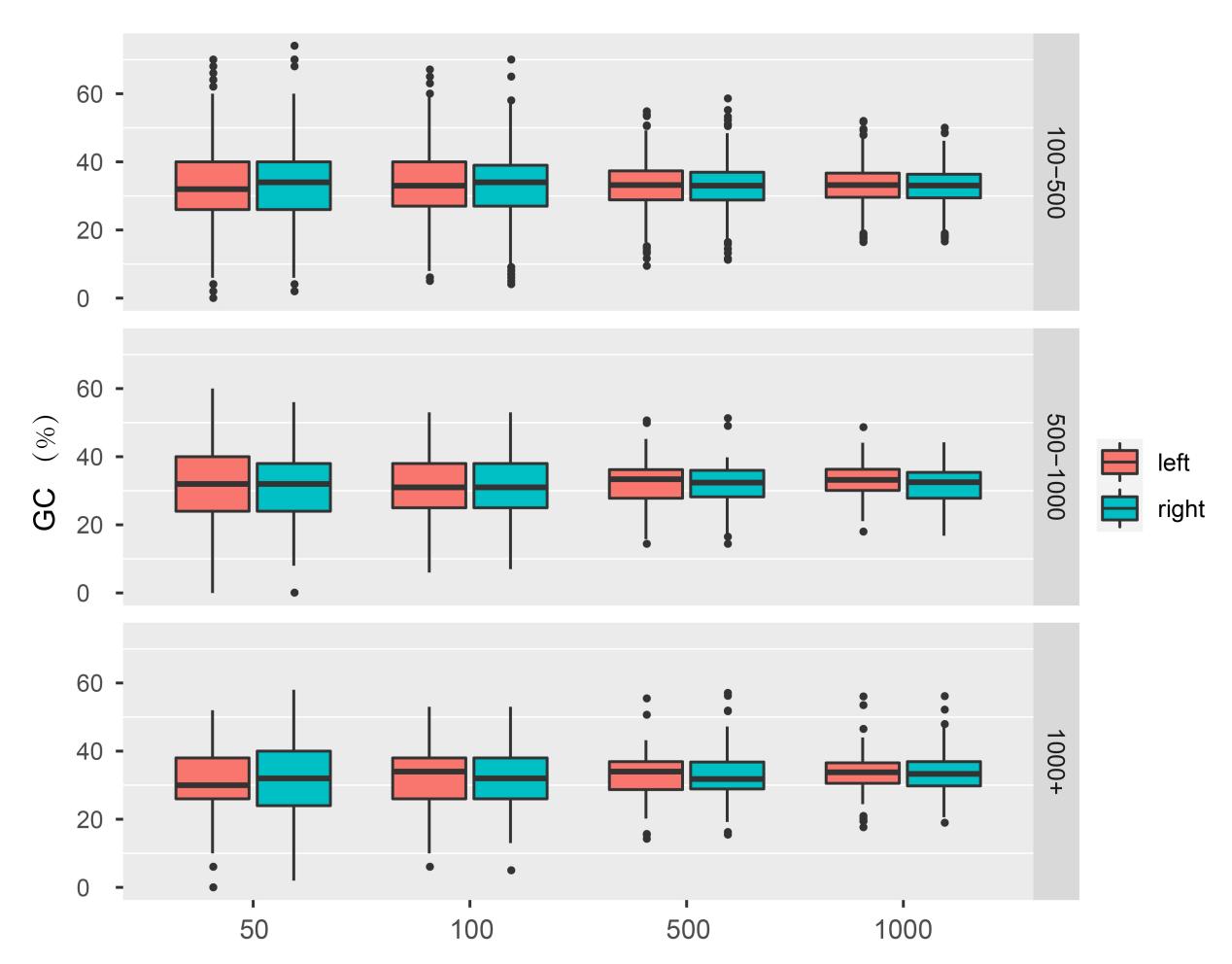


**Figure S9.** The GC content of nuclear genome flanking sequences adjacent to inserted mitochondrial fragments.


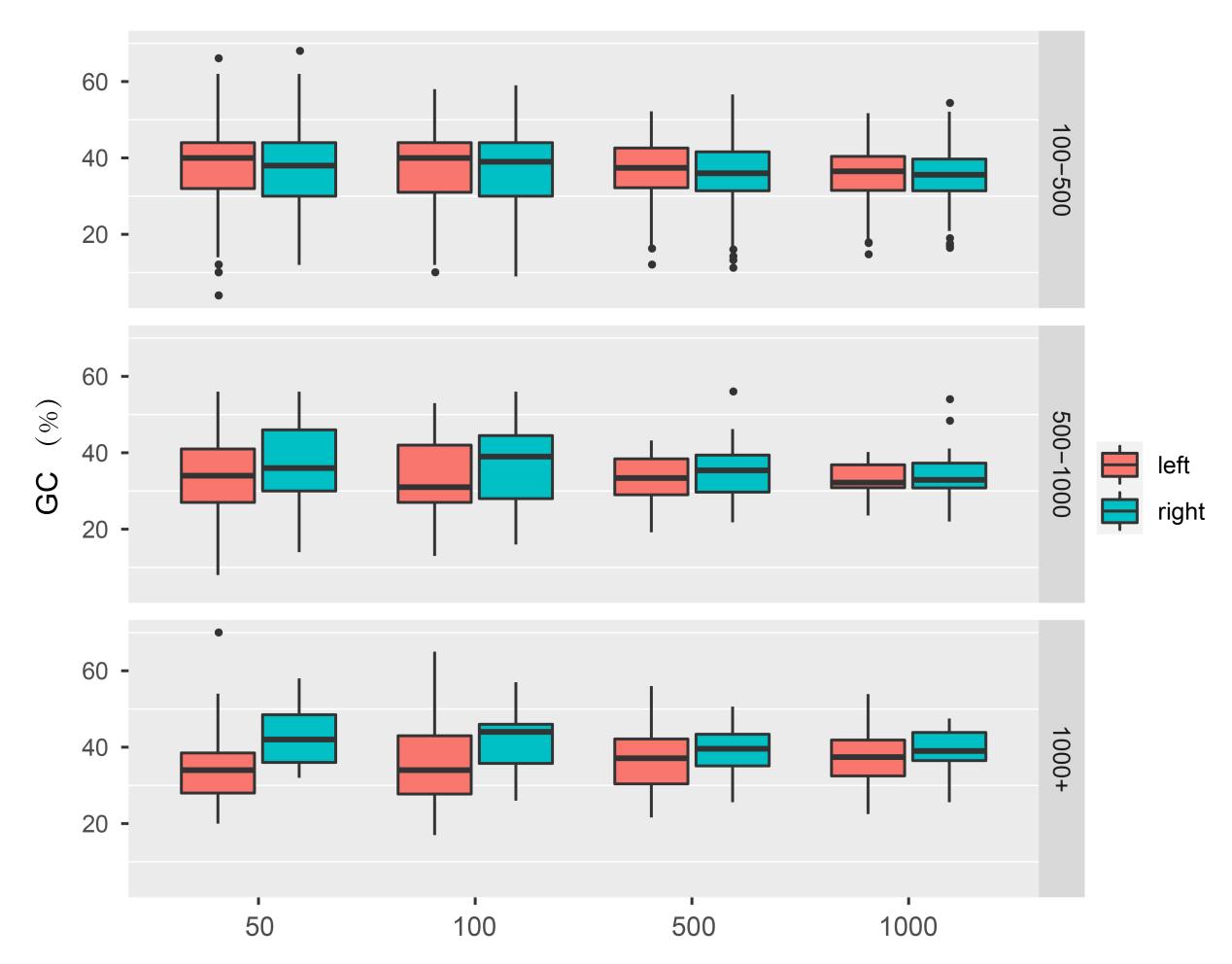


**Figure S10.** Dot-plot graphs indicating collinearity of chloroplast genomes in Faboideae as compared with *Styphnolobium japonicum* for reference.


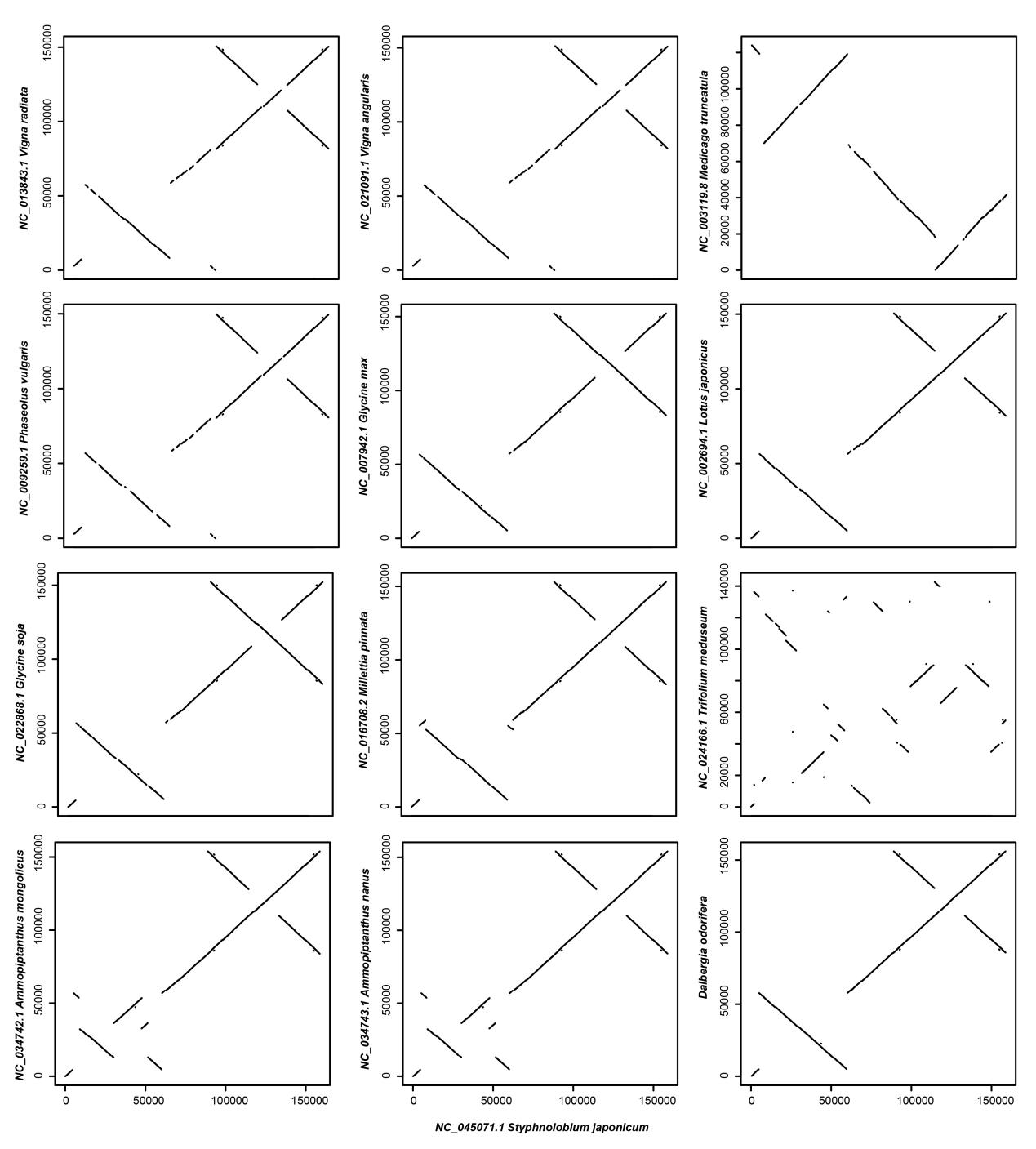


**Figure S11**. The bayes phylogenic tree of Fabaceae by using the chloroplast genes.


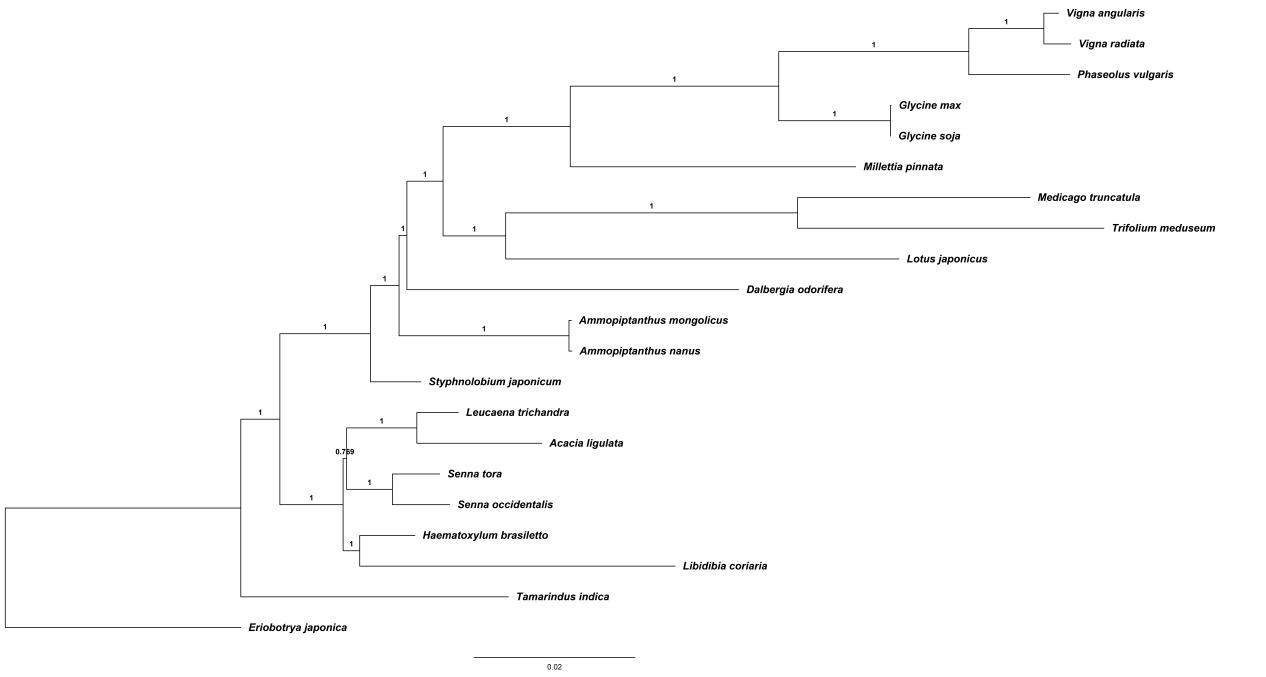


**Figure S12**. The bayes phylogenic tree of Fabaceae by using the mitchondrial genes.


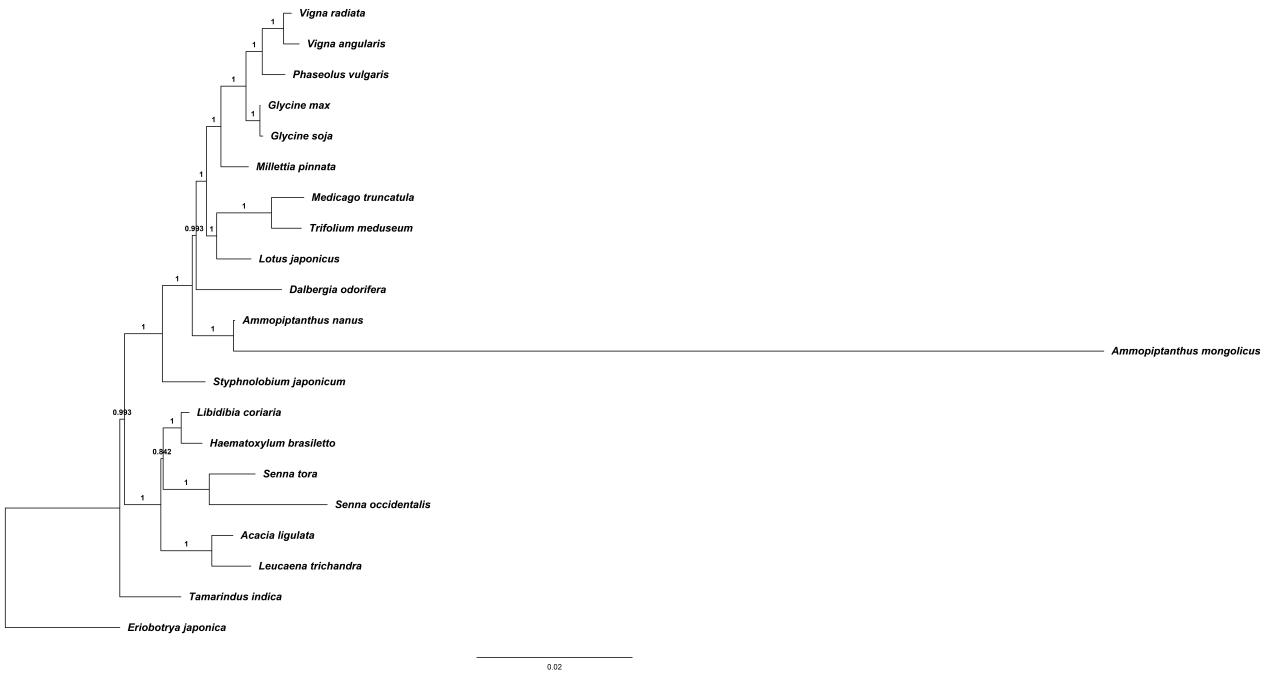


**Figure S13**. The phylogenic tree of Fabaceae chloroplast genes by using partition method.


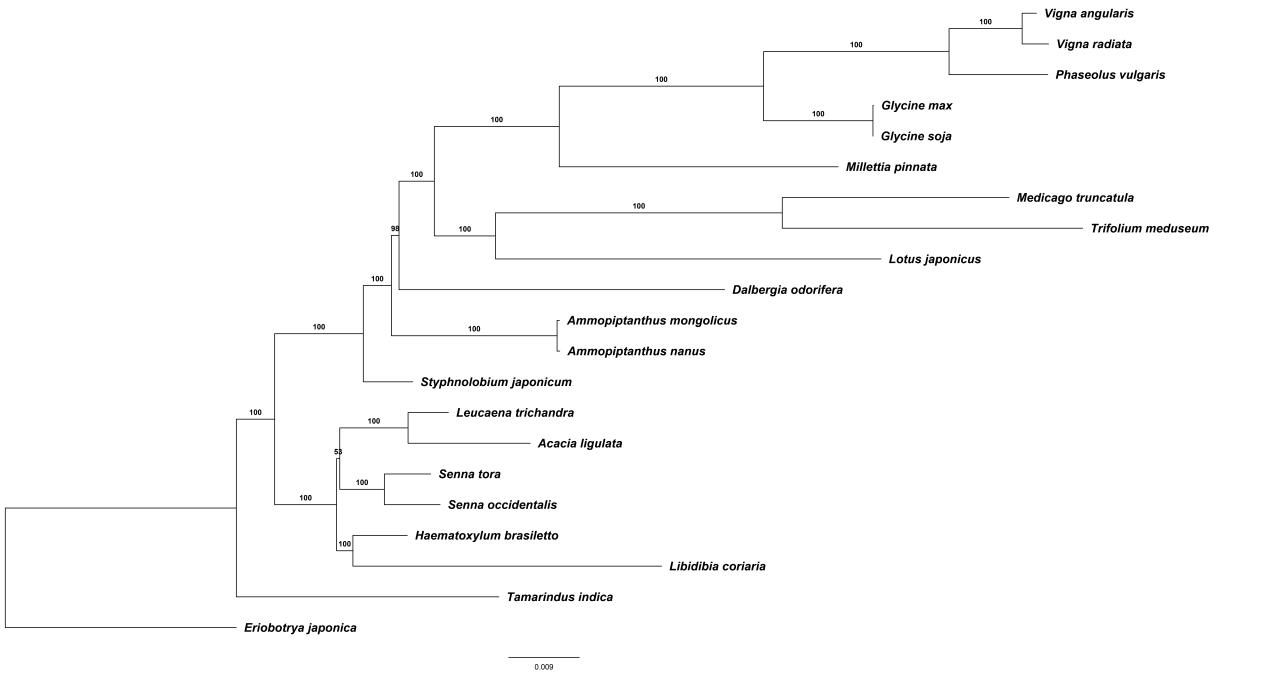


**Figure S14**. The phylogenic tree of Fabaceae mitchondrial genes by using partition method.


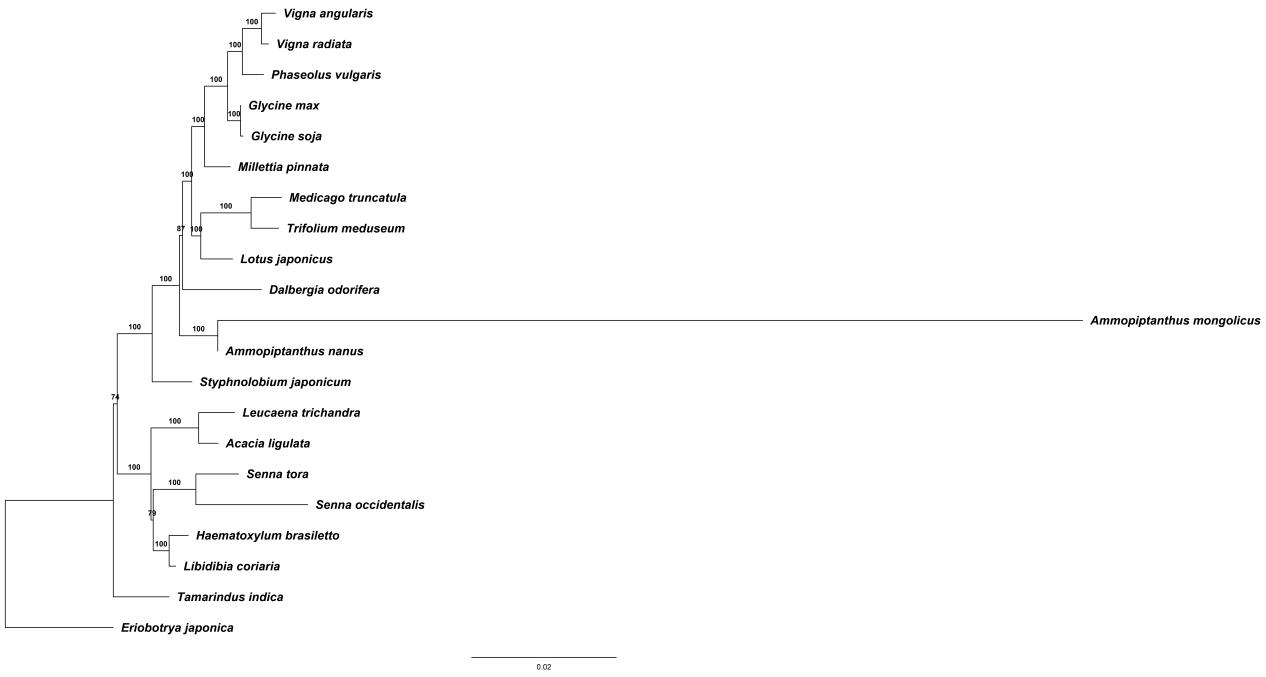

Supplement: Supplementary file 2 — Additional file 2: Fig. S1. Repeats found in the chloroplast genome of D. odorifera. Repeats detected are: F (forward direct match repeats), R (reverse match repeats), C (complement match repeats), P (palindromic match repeats). Fig. S2. Repeats found in the mitochondrial genome of D. odorifera. Repeats detected are: F (forward direct match repeats), R (reverse match repeats), C (complement match repeats), P (palindromic match repeats). Fig. S3. Dot-plot graphs indicating collinearity of mitochondrial genomes in Faboideae as compared with V. radiata for reference. Fig. S4. Dot-plot graphs indicating collinearity of mitochondrial genomes in Faboideae as compared with Ammopiptanthus nanus for reference. Fig. S5. The phylogenic relationships of Fabaceae organelles as inferred for chloroplast genes (left) and mitochondrial genes (right) with proportional branch lengths. Fig. S6. The percent of transferred sequence in each nuclear chromosome. Fig. S7. The frequency of organelle DNA transferred in each nuclear chromosome. Fig. S8. The GC content of nuclear genome flanking sequences adjacent to inserted chloroplast fragments. Fig. S9. The GC content of nuclear genome flanking sequences adjacent to inserted mitochondrial fragments. Fig. S10. Dot-plot graphs indicating collinearity of chloroplast genomes in Faboideae as compared with S. japonicum for reference. Fig. S11. The bayes phylogenic tree of Fabaceae by using the chloroplast genes. Fig. S12. The bayes phylogenic tree of Fabaceae by using the mitchondrial genes. Fig. S13. The phylogenic tree of Fabaceae chloroplast genes by using partition method. Fig. S14. The phylogenic tree of Fabaceae mitchondrial genes by using partition method. [file 12864_2021_7967_MOESM2_ESM.docx]
